# The relationship between marital status and cognitive impairment in Chinese older adults: the multiple mediating effects of social support and depression

**DOI:** 10.1186/s12877-024-04975-6

**Published:** 2024-04-24

**Authors:** Donghang Zhang, Wenhao Zheng, Keyang Li

**Affiliations:** 1 Department of Innovative Social Work, Faculty of Humanities and Social Sciences, City University of Macau, Macao, China; 2 Faculty of Humanities and Social Sciences, City University of Macau, Avenida Padre Tomás Pereira Taipa, 999078 Macao, China

**Keywords:** Marital status, Social support, Depression, Cognitive impairment, Chinese older adults

## Abstract

**Background:**

Marital status is a potentially essential factor for cognitive impairment. Relevant research examining the potential pathways through which the marital status of spouseless older people is associated with cognitive impairment needs to be more adequate. Therefore, this study aims to investigate the serial mediating effects of various forms of social support and depression between marital status and cognitive impairment in older Chinese people.

**Methods:**

This study involved a secondary analysis of data from the 2014–2018 wave of the Chinese Longitudinal Healthy Longevity Survey (CLHLS), with a total of 2,647 Chinese older adults and 53.6% being males. Mediation analysis using the SPSS process macro was conducted.

**Results:**

The results indicated that marital status was significantly predictive of cognitive impairment among older people, and those with a spouse exhibited higher cognitive functioning. Informal social support and depression were found to play partial mediating roles in the association between marital status and cognitive impairment. The findings also revealed that marital status was unrelated to formal social support, and no association between formal social support and cognitive impairment was found.

**Conclusions:**

The study findings highlight the need for social service providers to design programs for promoting connections associated with informal support to reduce their risk of depression and cognitive impairment and for policymakers to develop effective formal social support systems for older people without spouses. This study indicated that older people could regain the benefits of marriage to lower the risk of depression and improve their mental health.

## Introduction

Cognitive impairment is a chronic disease that occurs mostly in older adults and may eventually progress to dementia as the extent of the disease continues to deteriorate [1], which places various burdens on clients, caregivers, and the whole society. An estimated 10–15% of individuals living with mild cognitive impairment (MCI) develop dementia each year [2]. There are currently 15.07 million people with dementia and 38.77 million with mild cognitive impairment in China [3], and the number of people with dementia continues to rise. People also tend to be diagnosed with young-onset dementia at an early age [4]. There is no cure for dementia, and the mortality rate is high.

Implementing measures to mitigate risk factors associated with cognitive impairment can reduce the likelihood of developing dementia by 35% [5]. This has increased focus on exploring risk factors for cognitive impairment and dementia [6]. Marital status has been found to be associated with cognitive impairment and dementia risk, and older adults without a spouse have a higher risk of developing dementia [7]. Individuals who are long-term spouseless are more likely to develop cognitive impairment than those who have recently become spouseless [8]. A longitudinal qualitative study over 34 years indicated that the frequency of interaction with spouses increased from early adulthood onwards, with emotional closeness deepening throughout adulthood [9]. Spousal relationships and marital quality are influential in determining psychosocial and cognitive function, especially in older men [10, 11]. The enduring nature of marital status represents a potentially important yet often disregarded social factor that either elevates the risk or serves as a protective element against cognitive impairment in the long term [7]. Therefore, understanding the association between marital status and cognitive function has become a crucial aspect of exploring strategies to prevent cognitive impairment among older adults. Further research is warranted, especially those examining the potential pathways through which the marital status of long-term spouseless older people predicts cognitive impairment.

### Marital status and cognitive impairment

Compared to older people in stable marriages, those without spouses or experiencing marital dissatisfaction tend to receive less support, have a narrower social network, and experience a stronger sense of loneliness, which may influence the biological mechanisms that lead to cognitive decline and ultimately contribute to cognitive impairment in older people [12–14]. While having a spouse may offer important protection against cognitive impairment, the association between marital status and cognitive function among older adults varies across different countries. Studies indicated that being widowed was associated with significantly lower cognitive function relative to being married in China, but there is no difference between being married and being either never married or separated/divorced [15]. Typically, widowhood tends to happen later in life, compared to divorce [16]. More studies are needed to explore the relationship between marital status, particularly widowhood, and cognitive impairment in older Chinese people.

### The mediating effect of depression

Previous research has discussed the effects of marital status on depression symptoms in older adults [17, 18]. Marriage itself has unique social, psychological, and economic resources that are not available from other types of relationships, and these enabling factors are more likely to keep spoused older adults healthy and living longer [7]. Marital dissolution may lead to feelings of isolation, disapproval, lack of confidence in themselves, and reduced social activities, leading to depression in older people [12]. Moreover, depression was significantly related to cognitive functioning or impairment and increased mortality [19]. De Nooij et al. [20] proposed that depression and cognitive decline could co-occur and have a bidirectional relationship. According to research on older Chinese adults, severe depressive symptoms were linked to poorer cognitive performance in an elderly community [21]. However, less attention is given to the critical role of depression in this association between marital status and cognitive impairment.

### The mediating effect of social support

Marital status is also an essential factor in predicting social support among older adults [22]. Given that older people who are married and have higher-quality marital relationships benefit from greater caregiving and financial support provided primarily by their spousal caregivers, they may demonstrate a preferential reliance on informal social support. In contrast, never-married older people exhibit heightened dependence on non-spousal support [23], necessitating an orientation toward alternative forms of social support to meet their socio-emotional needs. Furthermore, social support was found to be associated with cognitive impairment [24]. In a systematic review of 22 articles published between 1999 and 2019 involving an empirical quantitative focus, Costa-Cordella [25] revealed a relevant positive association between social support and cognition. The cognitive reserve theory suggests that positive stimulating activities can alter the neural organization and foster new neural pathways, subsequently delaying cognitive decline [26]. Social support can promote social participation and interaction, leading to improved cognitive functioning [27]. Social support is divided into two forms: formal social support and informal social support. Further studies can examine how various forms of social support can mediate the association between marital status and cognitive impairment.

### Social support and depression

Evidence suggests that individuals with strong social support are likely to mitigate negative emotions [28], and compared to younger people, social support is more necessary for older people [29]. Among older adults, social support can significantly lower their risk of depression or alleviate depressive symptoms [30]. Formal social support has a buffering effect on older people’s mental health, implying lower rates of depression [31]. Meanwhile, informal social support, such as better child relationships, is closely associated with lower depression among older people [28]. Yang et al. [32] found that receiving emotional support from family members or a sex partner was associated with a reduced risk of depressive symptoms. Furthermore, depression may play a mediating role in the association between social support and cognitive impairment. Jin et al. [33] investigated 778 community-dwelling older adults over at least one year of follow-up and found that depressive symptoms could mediate the relationship between social support and frailty, and frailty was proven to increase the risk of future cognitive decline [34].

As older adults age, their social contact becomes narrower, and interpersonal communication among family members becomes the main form of social interaction [9, 26]. Older people without spouses who may lack emotional and instrumental support from their spouses and other family members in their lives are more likely to experience feelings of loneliness and are more susceptible to self-neglect and a tendency to isolate themselves within their homes [12, 35]. Long-term loneliness may lead to depression [36], manifesting in the symptoms of a decline in performing activities of daily living and social contact, which eventually hinder their ability to recognize and address personal health issues promptly and increase their vulnerability to the risk of cognitive impairment in older people [37]. Previous studies have identified the effects of social support and depression on cognitive impairment in developed countries [38, 39]. However, researchers have rarely discussed how marital status affects cognitive impairment by reducing social support and increasing depression among older adults from developing countries.

### The current study

This study aims to investigate the interlocking factors of cognitive impairment in China, examining the serial multiple mediating effects of various social support and depression between marital status and cognitive impairment in older Chinese adults. This study provides implications for exploring scientific and effective preventive measures to improve the well-being of older adults. Testing the relationship allows us to have a more comprehensive examination of how marital status contributes to the development of cognitive decline. In this study, we hypothesize the following: (1) there is a relationship between marital status and cognitive impairment in older adults; (2) formal and informal social support and depression mediate the association between marital status and cognitive impairment, respectively; and (3) formal and informal social support and depression have a serial mediation effect between marital status and cognitive impairment.

## Methods

### Data source and samples

This study was conducted with 2014 and 2018 data drawn from the Chinese Longitudinal Healthy Longevity Survey (CLHLS) project. CLHLS is a longitudinal survey of older people and the National Development Research Institute in China organized by the Center for Healthy Aging and Development Studies (CHADS) of Peking University, which was first conducted in 1998. Based on the baseline survey, CLHLS conducted the following seven surveys in 2000, 2002, 2005, 2008–2009, 2011–2012, 2014 and 2017–2018. The sampling design of CLHLS adopted a multistage disproportionate and targeted random sampling method, and the participants of this project were older people over 65 years old and their adult children aged 35–64 years in 23 Chinese provinces [40, 41]. The CLHLS investigated information on the health, socioeconomic characteristics, family background, psychological characteristics and lifestyle, and demographics of older Chinese people.

Given that the social support, depression, and cognitive impairment of older people are generally not immediately affected after divorce or widowhood, after four years, the social support, depression, and cognitive impairment measured in 2018 have become more stable. As such, to assess the role of the marital status of older people, this study included older people whose marital status was “whether married and living with spouse” using the 2014 data; social support, depression state, and cognitive impairment state were measured using the 2018 data, and new samples added in 2018 were excluded from this study. Additionally, we reclassified 8 participants who were consistently unmarried but living with their partners from 2014 to 2018 as having a spouse. In the process, we excluded 289 participants who were unmarried but either started or stopped living with a partner during this period because we could not determine the exact timing of these cohabiting arrangements. Eventually, 2,647 samples participated in both the 2014 and 2018 surveys, and their marital status remained unchanged.

### Measures

Cognitive impairment as an outcome variable was assessed using the Chinese version of the modified Mini-Mental State Examination (MMSE), a widely reliable tool for screening cognitive impairment internationally [42]. The scale includes six dimensions: general ability, reaction ability, attention and calculation ability, recall ability, language understanding and self-coordination ability, and cognitive function. The total score ranged from 0 to 30, with higher scores indicating better cognitive functioning. The internal consistency of the MMSE in this study was 0.73.

The marital status of older people as an independent variable was measured by asking whether they were married, divorced, widowed, or never married (with a spouse = 1, without a spouse = 0).

Social support as a possible mediator in this study includes formal and informal social support. Formal social support was evaluated based on older people’s access to social medical insurance and the number of services provided by the communities to older people. If older people participated in any public medical insurance (including urban workers’/residents’ medical insurance or new rural cooperative medical insurance), they were entitled to medical insurance and were assigned a value of 1; otherwise, they were assigned 0. The number of social services provided to older people in the community, including personal care, home delivery of medication and medical appointments, daily shopping, and nine other items, were summed. The total score of formal social support is the sum of the social health insurance and the community service scores. Informal social support was evaluated based on the children’s visits, contact, and financial support. One point was given if children visited often, 0 points otherwise; 1 point was given if children communicated often, 0 points otherwise; 1 point was given if children had given cash (including in-kind) support to older people in the previous year, 0 points otherwise. Higher scores indicate a higher level of social support.

Depression as the other possible mediator was measured using the simplified version of the Center for Epidemiologic Studies Depression Scale (CES-D) used in the CLHLS [43]. The scale uses a 5-point Likert scale with ten items. The answers ranged from 1 (always or very good), 2 (often or good), 3 (sometimes or fair), 4 (rarely or bad), to 5 (never or very bad). The total depression score was summed, with higher scores indicating a lower level of depression. The internal consistency of the depression scale in this study was.79.

Previous studies have indicated that gender and education are risk factors affecting cognitive impairment in older people [44, 45]. Therefore, gender and education were considered control variables (gender: male = 1, female = 0; education: years of schooling). CLHLS asked respondents about their gender and the school years they attended.

## Data analyses

The analysis of data for this study consisted of (1) descriptive analysis used to describe the general characteristics of the study population; (2) bivariate correlation analysis (t-test and Pearson’s r) to examine relationships between demographics and social support, depression, and cognitive impairment; and (3) a mediation analysis to explore the multiple mediating effects of social support and depression on the association between marital status and cognitive impairment. Descriptive data analysis, bivariable analysis, and mediation analysis were conducted using SPSS software, and the mediation analysis was performed using the Hayes SPSS macro PROCESS Macro program (Model 80) [46].

## Results

### Characteristics of samples

As shown in Tables 1, 53.6% of all participants were males (*n* = 1418). The average age was 85.46 ± 9.58 years old. Half of the participants (52.4%) had no education, and 58% reported no spouse. More than half of the older people reported that they were in very good or good health (48.4%), 39.3% reported an average healthy level, and 12.3% reported bad or very bad health.

**Table 1:** Demographic variables

Demographic variables		Number	Percent/%
Gender			
	Male	1418	53.6
	Female	1229	46.4
Education			
	Have no education	1372	52.4
	Have received education	1246	47.6
Marital status			
	No spouse	1535	58.0
	Have spouse	1112	42.0
Health status			
	Very good	260	10.1
	Good	991	38.3
	Average	1017	39.3
	Bad	290	11.2
	Very bad	27	1.1

Note: Missing values in education and health status are not included

### Correlation between major variables

As shown in Table 2, the results of the independent t-test showed significant differences between marital status and informal support (t=-2.92, *p* <.01), depression (t=-10.07, *p* <.001), and cognitive impairment (t=-15.46, *p* <.001). Sex differences were found in informal support (t = 2.25, *p* <.05), depression (t=-6.09, *p* <.001) and cognitive impairment (t=-10.45, *p* <.001). These findings indicated that older people without a spouse and females were more likely to have higher levels of depression and cognitive impairment, and older people with a spouse were more likely to have more informal social support.

**Table 2:** T tests for major variables

Variables		Formal social support		Informal social support		Depression		Cognitive impairment	
		*M* ± SD	*t*	*M* ± SD	*t*	*M* ± SD	*t*	*M* ± SD	*t*
Marital status	No spouse	2.65 ± 2.29	0.32	2.51 ± 0.76	-2.92^**^	34.38 ± 8.97	-10.07^***^	18.08 ± 5.84	-15.46^***^
	Have spouse	2.62 ± 2.24		2.59 ± 0.61		37.66 ± 7.13		20.99 ± 3.48	
Sex	Male	2.68 ± 2.34	-1.01	2.51 ± 0.72	2.25^*^	36.89 ± 7.74	-6.09^***^	20.44 ± 4.48	-10.45^***^
	Female	2.59 ± 2.21		2.57 ± 0.69		34.85 ± 8.79		18.34 ± 5.53	

Note: *p <.05, ** *p* <.01, *** *p* <.001

In Table 3, the results of Pearson’s correlation analysis revealed statistically significant associations between years of schooling, informal social support, formal social support, depression, and cognitive impairment, except for the link between informal and formal social support, informal social support, and years of schooling. Specifically, education was positively correlated with formal social support (*r* =.04, *p* <.05), depression (*r* =.21, *p* <.001), and cognitive impairment (*r* =.28, *p* <.001). High levels of formal social support and informal social support significantly reduce cognitive impairment, with correlation coefficients of 0.05 (*p* <.05) and.07 (*p* <.01), respectively. In addition, high levels of formal social support (*r* =.10, *p* <.001) and informal social support (*r* =.07, *p* <.001) significantly decrease depression. Finally, depression positively correlated with cognitive impairment (*r* =.36, *p* <.001).

**Table 3:** Pearson’s bivariate correlations between mediators and cognitive impairment

	M (SD)	Years of schooling	Formal social support	Informal social support	Depression	Cognitive impairment
Years of schooling	2.63 ± 3.55	1.00				
Formal social support	2.64 ± 2.27	0.04^*^	1.00			
Informal social support	2.54 ± 0.70	-0.03	-0.01	1.00		
Depression	35.83 ± 8.36	0.21^***^	0.10^***^	0.07^***^	1.00	
Cognitive impairment	19.34 ± 5.16	0.28^***^	0.05^*^	0.07^**^	0.36^***^	1.00

Note: *p <.05, ** *p* <.01, *** *p* <.001

### Mediation analysis of social support and depression

**Fig. 1:**
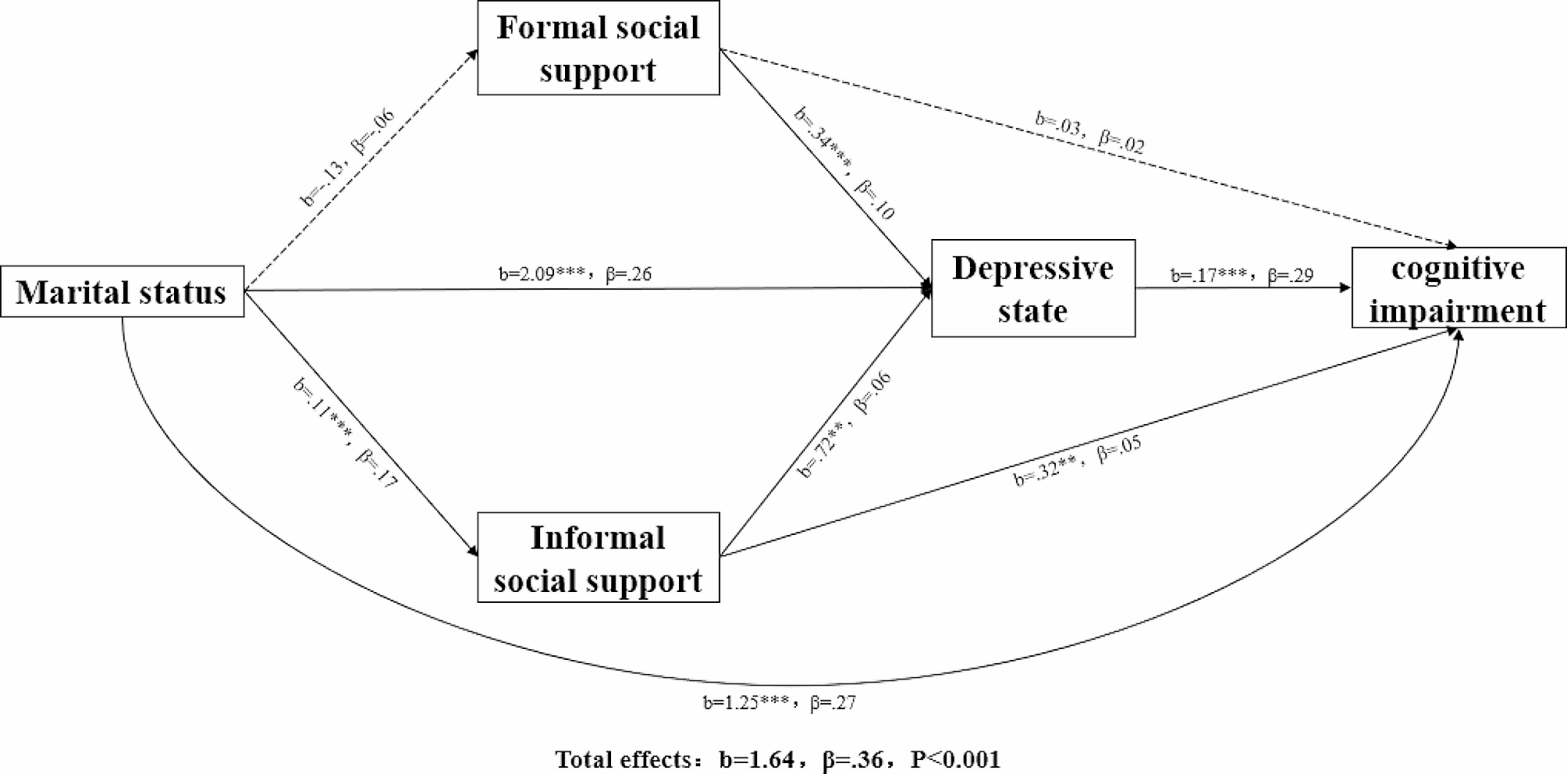
Results of multiple mediation analysis for cognitive impairment. (Note: *p <.05, ** *p* <.01, *** *p* <.001. Solid lines represent significant paths, while dashed lines represent a non-significant path)

The Hayes SPSS Process Macro using a bootstrapping method was employed to explore the serial mediating effects of social support and depression on the association between marital status and cognitive impairment. As shown in Fig. 1, the total effect of marital status on cognitive impairment was significant (b = 1.64, β = 0.36, *p* <.001). The results also revealed significant direct effects of marital status on informal social support (b = 0.11, β = 0.17, *p* <.001), depression (b = 2.09, β = 0.26, *p* <.001), and cognitive impairment (b = 1.25, β = 0.27, *p* <.001). The direct effect of marital status on cognitive function accounted for 76.22% (1.25/1.64) of the total variance in cognitive impairment. Informal social support was also found to be associated with depression (b = 0.72, β = 0.06, *p* <.01) and cognitive impairment (b = 0.32, β = 0.05, *p* <.01), and depression was positively predictive of cognitive impairment (b = 0.17, β = 0.29, *p* <.001). Formal social support was not associated with marital status or cognitive impairment, except for depression (b = 0.34, β = 0.10, *p* <.001). Second, for control variables, gender was associated with cognitive impairment (b = 0.62, β = 0.07, *p* <.01), as were years of schooling (b = 0.18, β = 0.15, *p* <.001).

As shown in Table 4, the 95% confidence interval (CI) of the indirect effect between marital status and depression through the mediating effect of informal social support and between indirect and cognitive impairment through the mediating effect of depression did not include zero, indicating that the indirect effect was significant (indirect effect of informal social support = 0.04, indirect effect of depression = 0.34). Depression, serving as a mediator between marital status and cognitive impairment, explained 20.73% (0.34/1.64) of the variance in cognitive impairment. Informal social support, serving as a mediator between marital status and cognitive impairment, explained 2.44% (0.04/1.64) of the variance in cognitive impairment. The third indirect pathway was that the effect of marital status on cognitive impairment was significantly mediated by both informal social support and depression, with an indirect effect value of 0.01. The chain mediation of informal social support and depression explained 0.61% (0.01/1.64) of the variance in cognitive impairment. As such, the result of the bootstrap test confirmed the existence of a partially mediating effect for informal social support and depression between leisure marital status and cognitive impairment.

**Table 4:** Indirect effects of mediation analysis of marital status on cognitive impairment

Relationships	B	BootSE	β	LLCI	ULCI
Indirect effects					
Marital status → formal social support →cognitive impairment	-0.01	0.01	-0.01	-0.01	0.01
Marital status → informal social support →cognitive impairment	**0.04**	**0.02**	0.01	0.01	0.08
Marital status → depressive state →cognitive impairment	**0.34**	**0.07**	0.07	0.22	0.49
Marital status → formal social support → depressive →cognitive impairment	-0.01	0.01	-0.01	-0.02	0.01
Marital status → informal social support → depressive →cognitive impairment	**0.01**	**0.01**	0.01	0.01	0.03

Note: Standardized estimates of 5,000 bootstrap samples. Bold indicates statistically significant findings. LLCI: lower level of the 95% confidence interval; ULCI: upper level of the 95% confidence interval. *p <.05, ** *p* <.01, *** *p* <.001

In other words, older people with a spouse are likely to receive more informal social support, leading to a lower level of depression and, eventually, a reduced level of cognitive impairment compared with older people without a spouse. The 95% CI of the indirect effect through formal social support includes zero, indicating that no indirect effect was found.

## Discussion

This study examined the relationship between marital status and cognitive impairment and the mediating role of social support and depression between the two. A potential pathway from marital status to cognitive impairment was identified, with high levels of informal social support associated with low levels of depression, highlighting the importance of constructing effective informal social support. This meant that older adults without a spouse had less informal social support, more severe depression, and poorer cognitive function. Although marital status did not significantly predict the formal social support of older adults, formal social support still significantly predicted depression and was indirectly associated with the cognitive impairment of older people through the mediating effect of depression. Furthermore, in our conceptual model, depression is the most immediate predictor of cognitive impairment, which was confirmed in previous empirical studies [20, 39]. Zhou et al. [21] found that severe depressive symptoms are associated with worse cognitive states in older Chinese adults.

The results of our study showed that marital status was significantly predictive of cognitive impairment in older people, accounting for more than 3/4 of the total variance in cognitive impairment. This finding is consistent with previous studies [7, 12], with those having a spouse exhibiting higher cognitive functioning. The ‘marriage protection effect’ highlights that being married can affect the health level and longevity of individuals by providing emotional support and related social support from their spouses and promoting a healthy lifestyle [47], indicating that marital status enhances cognitive functioning in older people. This finding also supports the marriage resource model, which proposes that marriage offers multiple forms of resources for individuals with a spouse [48]. Furthermore, research has indicated that people with a spouse are more inclined to access better medical resources, receive higher-quality medical care services, and benefit from various types of preventive care [49]. Their spouses can assist in monitoring unhealthy behaviors in older people, thereby reducing the occurrence of smoking, alcoholism, and other health-damaging behaviors [50]. Conversely, marital breakdown due to divorce or widowhood may lead to financial and emotional distress, directly impacting cognitive functioning [51].

Our study found that formal social support could not mediate the association between marital status and depression and cognitive impairment since marital status was not related to formal social support. This finding is consistent with Fu and He’s [52] study, which determined that marital status did not impact older people’s perceived level of formal social support. In other words, being married or not may not affect individuals’ access to formal social support. Instead, the most influential factors that predict formal social support are the difficulties and vulnerabilities older adults face [52].

No association between formal social support and cognitive impairment was found, which implies that formal social support in this study may not be sufficient to confer a protective effect on older people’s cognition. Cognitive impairment is an implicit, neglected health issue in China, and the measure of formal social support used in this study does not reflect the extent to which social service provisions are linked with cognitive impairment. Furthermore, in China, formal social support for older people in less developed areas is severely lacking, with the provision of quality formal social services falling short of what is needed [53]. The amount of formal financial support is relatively small and fails to meet the needs of older people with low household incomes, especially those from rural areas. This insufficiency in covering their consumption and medical expenditures may contribute to accelerating their cognitive decline [52].

Furthermore, the current social security offerings in China may not be sufficient to mitigate the risk of cognitive disorders in older adults [54]. Unlike in the U.S., the consumption expenditures of older people in China are primarily directed toward essential goods [55]. Their spending is relatively homogeneous and health care awareness is weak. When older individuals face severe or unforeseen health problems, formal social security support and services are inadequate. This is reflected in the high threshold for medical reimbursements and the disparity in healthcare services between urban and rural areas [45]. Moreover, resources for mental well-being are insufficient, not only for older people in underdeveloped regions but also in small and medium-sized cities. This gap is attributed to the lack of adequate focus on preventing and treating cognitive disorders among older people, making it challenging to offer one-on-one assistance to older individuals living alone through resource mobilization either in village or urban community committees [56]. As such, no relationship was observed between formal social support and cognitive impairment in this study. However, formal social support can positively prevent depression among older people [57], underscoring the importance of social services provided to older people in reducing depressive symptoms.

In addition, our findings indicated that informal social support mediated the association between marital status and depression, which is in line with previous research [28, 52]. Chinese cultural traditions value family unions and bonds, and informal social networking is vital for connecting family members. Older people are more likely to be eager for companionship and interactions with family members to eliminate their feelings of loneliness that are closely related to depression [58], especially those living without a spouse and with insufficient social resources.

The key finding in this study is that informal social support mediates the relationship between marital status and cognitive impairment in older people, which is echoed in other studies [59]. Offspring visits were consistently associated with a lower incidence of cognitive impairment in older Chinese adults [60]. Emotional support from family members significantly contributes to the prevention of further physical and cognitive deterioration and helps mitigate cognitive decline [59]. These findings support the positive association of informal social support with cognitive function in older people. Although the effect size of informal social support as a mediator is small, it can not only compensate for the limitations of social security systems but also serve as a substitute for the inadequacy of professional care services. This highlights the negative effect of the absence of a spouse on the availability of informal social support and the critical role of informal social support as a determinant of older adults’ health, including depression, cognitive functioning, and other health indicators.

In line with previous research findings [12, 61], this study also indicated that depression mediated the association between marital status and cognitive impairment in older people. The indirect effect of this pathway, explaining 1/5 of the variance, underscores the pivotal role of depression in the link between marital status and cognitive function. Adverse life events such as separation, divorce, and spousal death are predictive of depression [62], which in turn contributes to cognitive impairment [63]. Additionally, our study confirmed the serial mediating effects of informal support and depression on the association between marital status and cognitive impairment. This suggests that older adults with a spouse may receive more informal social support, leading to a reduced risk of depression and, eventually, a lower risk of cognitive impairment. This finding is consistent with the findings of previous studies [33, 64].

Depression fully served as a mediator in the relationship between formal social support and cognitive impairment in this study, while it played a partial mediating role in the relationship between informal social support and cognitive impairment. This finding revealed the differential role of informal and formal social support. Previous studies have emphasized the importance of informal social support, but formal social support can also contribute to reducing depression and enhancing cognitive function in older people, which can be considered a valuable complement to informal social support.

### Limitations

This study has two limitations. First, since this study entailed secondary data analysis of the CLHLS data, accumulated measures may not capture the validity of formal and informal social support. For example, formal social support was measured by access to social medical insurance and summing up the number of social services provided to older people. Adopting a standardized measure of social support would strengthen this study. Second, the use of cross-sectional data from 2018 to assess social support, depression, and cognitive impairment imposes constraints in deriving causal relationships. While the longitudinal association between marital status and cognitive impairment may vary according to gender and across different levels of cognitive function [10], other research has indicated cognitive impairment may be related to marital status [65, 66], as well as to depression [67]. Furthermore, depression has been shown to diminish an individual’s social support [68]. Future studies should investigate the long-term effects of marital status on cognitive impairment through social support and depression, as well as consider the potential impact of confounding factors and reverse causation among these variables.

### Implications

This study has several implications for developing older people’s cognitive health. Echoing most previous studies’ findings [69], older people without a spouse had significantly higher depression and cognitive impairment than those with a spouse, and they also had significantly lower informal support than those with a spouse. This observation highlights the need for increased attention to older people without spousal companionship, as they are at a higher risk for depression and cognitive impairment. Social support was found to alleviate depression and cognitive impairment in older adults. These findings highlight the need for policymakers to develop comprehensive, high-coverage health insurance and person-centered approach care systems for older people without spouses and for social service providers to design social service programs to reduce their risk of depression and cognitive impairment. In addition, these findings support the assertion that financial and emotional support from family members is important for older people without spouses. Encouraging spouseless older adults to find a spouse is also a practical approach [70], which helps older adults regain the benefits of marriage, lowers their risk of depression, and improves their mental health. Most importantly, this study emphasizes the importance for social workers to develop psychosocial interventions for lowering depression risk among older people.

## Conclusions

Less attention is given to examining the potential pathways through which marital status affects cognitive impairment among older people. Evidence from a National sample of older adults in China indicated the effect of marital status on cognitive impairment by reducing social support and increasing depression among older adults. The key finding in this study has revealed that informal social support significantly contributes to mitigating depression and cognitive decline in older adults without spouses, and financial and emotional support from family members is essential.

## Data Availability

The data that support the findings of this study are available at https://opendata.pku.edu.cn/dataset.xhtml? persistentId=doi:10.18170/DVN/WBO7LK.

## Abbreviations

CLHLS: Chinese Longitudinal Healthy Longevity Survey
MMSE: Mini-Mental State Examination
CES-D: Center for Epidemiologic Studies Depression Scale
